# Impact of alternate partial root-zone irrigation on the rhizosphere microbiota of alfalfa plants inoculated with rhizobia

**DOI:** 10.3389/fmicb.2024.1372542

**Published:** 2024-07-10

**Authors:** Junhong Zou, Jianhui Xin, Tiemei Wang, Qing Song

**Affiliations:** ^1^School of Grassland Science, Beijing Forestry University, Beijing, China; ^2^Inner Mongolia Horqin Grassland Ecosystem National Observation and Research Station, Inner Mongolia, China

**Keywords:** alfalfa (*Medicago sativa*), drought stress, alternate partial root-zone irrigation, microbial biomass, microbial species abundances

## Abstract

Water is an important constraint on alfalfa (*Medicago sativa*) production in arid and semiarid areas, and alternate irrigation in root areas has water-saving potential for alfalfa production. To investigate the impact of alternate partial root-zone irrigation (APRI) on the rhizosphere soil microorganisms of alfalfa, this study subjected alfalfa plants to different irrigation methods and irrigation levels. The growth status and rhizosphere soil microbial community diversity of alfalfa plants under alternate root-zone watering treatment were analyzed through laboratory experiments and high-throughput sequencing. The results showed that at soil moisture levels of 80% field moisture capacity (FMC) and 60% FMC, APRI had no significant impact on the biomass or nodule number of alfalfa. However, 40% FMC significantly reduced the individual plant dry weight, chlorophyll content, and nodule number of the alfalfa plants. APRI increased the relative abundance of Actinomycetes in the alfalfa rhizosphere soil. Moreover, at 60% FMC, the MBC and MBN of rhizosphere, relative abundance of Actinobacteria and unclassified K fungi and Chao 1 index of bacteria significantly increased under APRI treatment. While relative abundance of Ascomycetes and Proteobacteria in the alfalfa rhizosphere significantly reduced under 60% FMC + APRI treatment. In summary, under the same irrigation conditions, APRI did not significantly affect the growth of alfalfa in the short term. And 60%FMC + APRI treatment did significantly affect the groups, structure and diversity of the rhizosphere soil microbial communities.

## 1 Introduction

Alfalfa is a perennial leguminous forage plant known for its good palatability, high yield, high nutritional value, and strong resistance to adverse conditions, making it an important cultivated forage plant worldwide (Guo et al., 2019). Drought is one of the major limiting factors leading to a decrease in agricultural productivity and slow crop growth globally (Anjum et al., 2017). Alfalfa is highly dependent on water resources, and its photosynthesis and metabolism are affected by water availability (Schimel et al., 2007; Jaleel et al., 2009). Water strongly influences the yield and quality of alfalfa plants. Most related research has suggested that adding water reduces the stem–leaf ratio of alfalfa and improves its quality (Lu et al., 2020). Adequate water is the basis of high yield and high-quality alfalfa production, but the alfalfa planting areas in China are mostly arid and semiarid areas, and arid climates, scarce precipitation and scarce water resources are the main factors limiting alfalfa production in these areas.

The specific operation of alternate partial root-zone irrigation (APRI) in the root area is to normally irrigate some root areas during the whole or part of the plant growth period, while the other area is subjected to a certain degree of water stress, and the two root areas alternate after a period (Hui and Luo, 2021); this irrigation technique is also known as alternate root zone drying technology (Hu et al., 2009). The main features of this technique are water savings, low investment, and easy implementation (Wang et al., 2021). It has been widely applied in food and fruit crops in arid and semiarid regions globally (Jha et al., 2019). The effect of the APRI on crop biomass differs among studies. Several studies have indicated that sorghum crop biomass has no effect on sorghum production (Liu et al., 2017), while others have shown an increase or decrease in biomass (Khaleghi et al., 2020). These differences arise from variations in the research subjects, sampling times, and experimental methods. A study of the impact of the APRI on alfalfa revealed that increasing the path between fields via alternate irrigation improved the water use efficiency of alfalfa without reducing yield (Zhang et al., 2016, 2020, 2021). In a study on the response of the main functional traits of the alfalfa root system to alternate partial root zone irrigation, it was found that this irrigation method reduced the biomass and root diameter of alfalfa plants and increased the number of root branches (Liu L. X. et al., 2023).

Research on APRI has focused mainly on its effects on plant biomass, the root system, nutrient absorption and utilization, and soil nutrients. Studies on soil microorganisms, especially microbial diversity, are relatively limited. Most related research has focused on the impact of the APRI on soil microbial quantity, such as the effect of the APRI on the rhizosphere soil microorganisms of maize (*Zea mays*) and rice (*Oryza sativa*), which showed that the APRI increased the total number of microorganisms in the maize rhizosphere soil and the activity and quantity of bacteria and actinomycetes in the rice rhizosphere soil (Wang et al., 2008; Liu et al., 2014). Research has also indicated that the quantities of bacteria, fungi, and actinomycetes in maize rhizosphere soil under APRI treatment are greater than those under conventional irrigation (Yv et al., 2008). Soil microbial diversity is an important indicator for evaluating the stability of soil ecosystems (Li et al., 2019). Soil moisture conditions affect the diffusion and transformation of soil nutrients and significantly impact the soil microbial community structure (Manzoni et al., 2012; Kaiser et al., 2015). Studies on soil microorganisms under drought stress have focused mainly on uniformly watering the soil in the plant root zone. For example, uniform drought can alter the diversity of bacterial communities in the plant rhizosphere, enriching gram-positive bacteria and reducing gram-negative bacteria (Fuchslueger et al., 2014; Breitkreuz et al., 2021). Uniform drought rapidly increased the relative abundance of actinomycetes in the rhizosphere soils of sorghum (*Sorghum bicolor*), rice, and wheat (*Triticum aestivum*) (Santos-Medellín et al., 2017; Mickan et al., 2019; Wipf et al., 2021) and increased the relative abundance of the phylum Firmicutes in the rhizosphere soil of wheat (Mickan et al., 2019). Experimental results from southwestern Australia indicated that uniform drought did not significantly affect fungal diversity in rhizosphere soil (Hopkins et al., 2018).

Inoculation with rhizobia is a commonly used method to increase alfalfa production. The nitrogen-fixing ability of rhizobia symbiotically nodulating alfalfa provides them with some advantages in resisting water stress. Yang et al. (2013) reported that inoculation with rhizobia significantly enhanced the drought resistance of alfalfa, and research by Zhang et al. (2013) also indicated that alfalfa treated with rhizobia had a stronger osmotic regulation ability and exhibited strong drought resistance. Investigating the effects of drought on leguminous forage plants inoculated with rhizobial bacteria not only provides experimental conditions more closely resembling those of natural cultivation but also has practical significance for the actual production of leguminous forage plants. Therefore, this study used alfalfa as an example and, through indoor flowerpot experiments, investigated the effects of APRI on the rhizosphere soil microorganisms of alfalfa inoculated with rhizobia.

## 2 Materials and methods

### 2.1 Experimental methods

This research was conducted in an artificial climate chamber at the School of Grassland Science, Beijing Forestry University. The plant growth environment consisted of 16 h of light and 8 h of darkness, with a constant temperature of 28°C and ample illumination provided by light-emitting diode light. The soil used for the experiment was obtained from the BaJia Nursery of Beijing Forestry University and was characterized as loam with a pH of 8.76 and a field moisture capacity (FMC) of 18.1%. The other basic physical and chemical properties of the soil were as follows: bulk density, 1.51 g/cm^3^; total porosity, 38.1%; capillary porosity, 33.98%; noncapillary porosity, 4.12%; organic matter, 13.43 g/kg; total nitrogen, 0.53 mg/kg; alkaline nitrogen, 19.31 mg/kg; effective phosphorus, 13.23 mg/kg; and quick phosphorus, 151.91 mg/kg (Feiwei et al., 2020). The flowerpot height was 40 cm, and the side length was 20 cm, with each flowerpot containing 7 kg of soil. In the alternate irrigation treatment group, a plastic partition was used to divide the flowerpots into two impermeable sections, with an intermediate 2 cm long and approximately 1 cm deep groove.

The experiment commenced on February 20, 2023, when the alfalfa ‘Beilin 201’ was used. Five similar healthy alfalfa seeds were planted in each flowerpot. For flowerpots with a partition, the seeds were planted as close to the depression under the partition as possible. After the alfalfa plants sprouted their third main leaf, one vigorous and uniform plant near the center of each flowerpot was retained for inoculation with rhizobia. The rhizobium strain used was *Sinorhizobium meliloti* from the China General Microbiological Culture Collection Center (ACCC17650). The rhizobium strain was inoculated onto yeast mannitol agar [10 g mannitol, 0.5 g potassium dihydrogen phosphate, 0.2 g sodium sulfate, 0.05 g sodium chloride, 0.4 g yeast extract, 4 mL RH trace element fluid (H_3_BO_3_ 0.5 g/mL, Na_2_MoO_4_ 0.5 g/mL, and distilled water 100 mL) (Cao et al., 2023), 15 g agar, and distilled water to a volume of 1,000 mL] and cultured at a constant temperature of 28°C for 48 h in an incubator. After washing with sterile water, the rhizobia were transferred to sterile conical flasks and shaken to form a suspension. The concentration of rhizobia per alfalfa plant was determined to be approximately 1 × 10^6^ using a hemocytometer.

### 2.2 Experimental design

**Figure 1:**
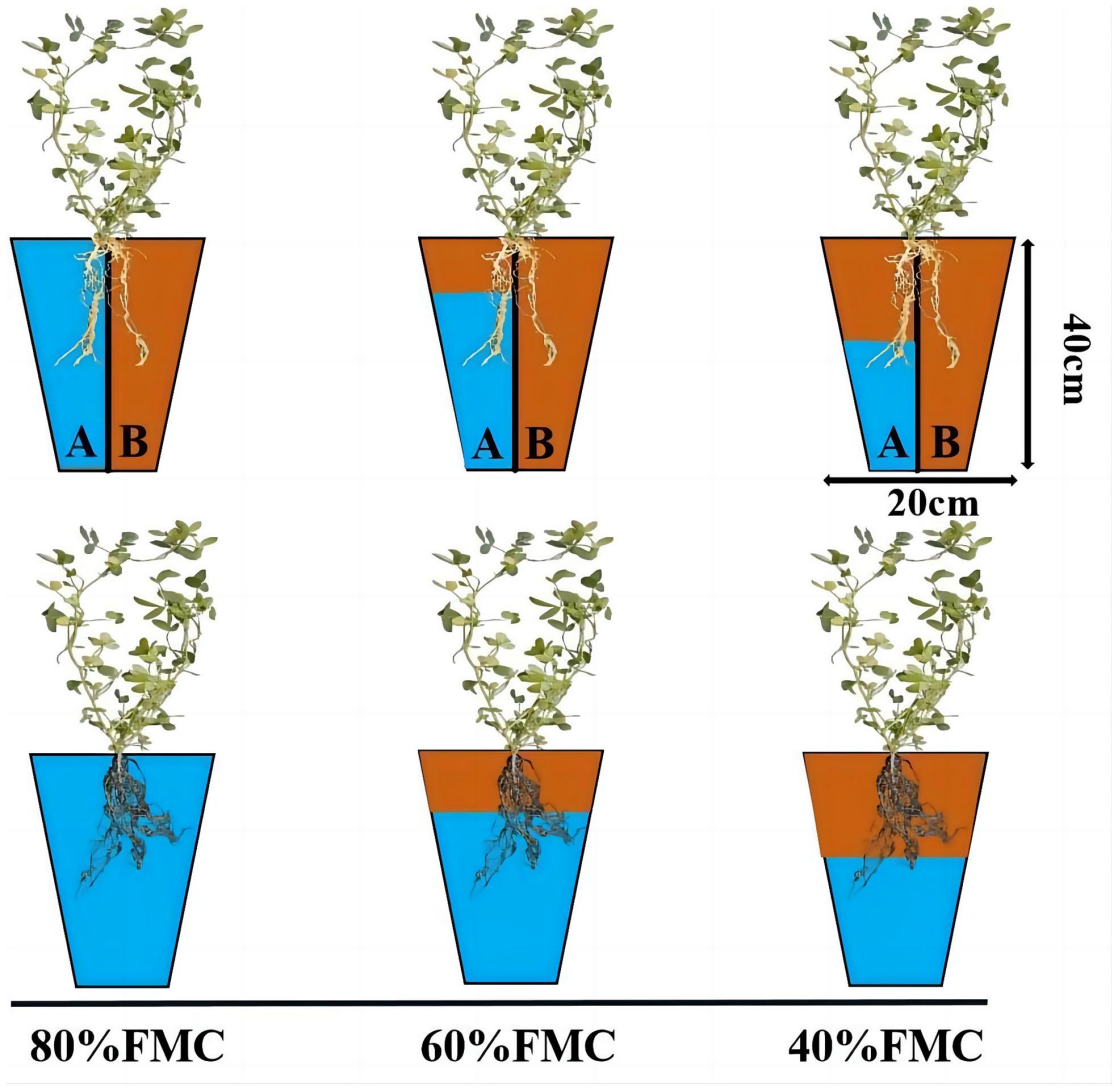
Schematic diagram of experiment design. The pots have partitions to indicate APRI treatment, and no partition indicates even watering treatment. The blue area indicates the amount of watering (80%FMC, 60%FMC, and 40%FMC).

After inoculation with Rhizobium, water control treatments were applied to the alfalfa plants. The experimental treatment design is shown in Figure 1. The water treatment regimens used were 80, 60, and 40% of the FMC, representing no drought, mild drought, and severe drought, respectively. The soil moisture content was controlled daily by weighing; the 80% FMC treatment consisted of a total of 7.39 kg (flowerpot, soil and water); the 60% FMC treatment consisted of a total of 7.19 kg (flowerpot, soil and water); and the 40% FMC treatment consisted of a total of 6.99 kg (flowerpot, soil and water). The irrigation methods included even watering in the root zone and APRI. For the APRI treatment group, the sides of the flowerpots were labeled A and B, and alternate irrigation was applied. The irrigation volume was half of the reduced weight, and when the cumulative irrigation volume reached 200 mL on one side, the irrigation root zone was exchanged. Each treatment was repeated 3 times. During the experiment, the positions of the flowerpots were randomly adjusted every day to reduce long-term imbalances in light, temperature, etc., that could cause deviations. In this study, no fertilizers were applied to the alfalfa plants.

Samples from the early flowering stage of alfalfa were collected, and growth indicator levels were determined. Three mature leaves were randomly selected from each alfalfa plant, a TYS-4N4N handheld chlorophyll meter (TuoPuYunNong Science & Technology Co., Ltd., Zhejiang, China) was used to measure the chlorophyll content, and the average value was recorded. The leaves were cut for chlorophyll content measurement. A Yaxin-1241 leaf area meter was used to measure the leaf area. The average leaf area was calculated for each alfalfa plant, and the average value was recorded. Subsequently, the 3 cut leaves from each alfalfa plant were stored at 4°C, after which the intact root system was removed. The soil around the roots was gently removed, after which the soil from 0 to 3 cm around the roots of the alfalfa plants was collected from the same treatment, and the soil was stored at −80°C for later use. Afterward, the alfalfa roots were gently washed with tap water to remove soil particles, and the number of nodules was recorded. Then, the moisture from the roots of the alfalfa plants was removed with absorbent paper, and the entire alfalfa plant and the leaves used for area measurements were dried in an air-blowing thermostatic oven. After the drying step was completed, an electronic balance (0.0001 g) was used to weigh and record the dry weight of a single alfalfa plant.

Beijing Owesen Genetech Co., Ltd., analyzed the microbial biomass carbon (MBC) and microbial biomass nitrogen (MBN) content and assessed the bacterial and fungal diversity in the rhizosphere soil; the chloroform fumigation extraction method was used to determine the MBC and MBN (Li et al., 2008). An E.Z.N.A.^®^ Soil DNA Kit was used to extract genomic DNA, and a Nanodrop 2000 was used to measure the quality and concentration of the DNA. The universal primers 338F (5′-ACTCCTACGGGAGGCAGCAG-3′) and 806R (5′-GGACTACHVGGGTWTCTAAT-3′) were subsequently used to amplify the V3-V4 region of the microbial 16S rRNA genes. Fungal PCR amplification was performed using universal ITS primers (Internal Transcribed Spacer 1, ITS 1). The purified PCR products were subsequently utilized for library construction using the NEBNext Ultra II DNA Library Prep Kit. The constructed libraries were purified using an Agencourt AMPure XP nucleic acid purification kit. After quality control, the constructed library was sequenced on an Illumina MiSeq/Novaseq 6,000 platform via the PE250/PE300 sequencing strategy.

### 2.3 Data processing and analysis

The original data obtained from high-throughput sequencing were assembled, quality controlled, and filtered to remove chimeras, producing optimized sequences (Grice et al., 2009). Vsearch software was used for OTU clustering analysis, wherein sequences with a similarity greater than or equal to 97% were categorized into the same operational taxonomic unit (OTU). The representative sequences of the OTUs were compared with the Silva13 database using the BLAST algorithm, with an e-value threshold set at 1e-5 to obtain species classification information for each OTU. QIIME software was used to calculate alpha diversity indices based on OTUs and their abundances, and R language was used for visualization. Species composition bar chart analysis was conducted in R based on the species annotation and relative abundance results. Linear discriminant analysis effect size (LEfSe) analysis was further conducted with the following data analysis steps. First, analysis of variance (ANOVA) was used to detect the species, with the threshold set at 0.05. The group difference was determined with the threshold set at 0.05. Finally, linear discriminant analysis (LDA) was used to minimize the influence of species (i.e., the LDA score), with the threshold value set at 3.

To evaluate the effects of irrigation methods and soil moisture content, variance analysis was separately performed for all the data. The statistical analysis was conducted using Excel 2010. SPSS 22.0 software was used for variance analysis of the measured data (significance level *p* < 0.05 and *p* < 0.01); the average values and standard deviations of each index were used to represent the measurement results.

## 3 Results

### 3.1 The impact of APRI on the growth of alfalfa

**Figure 2:**
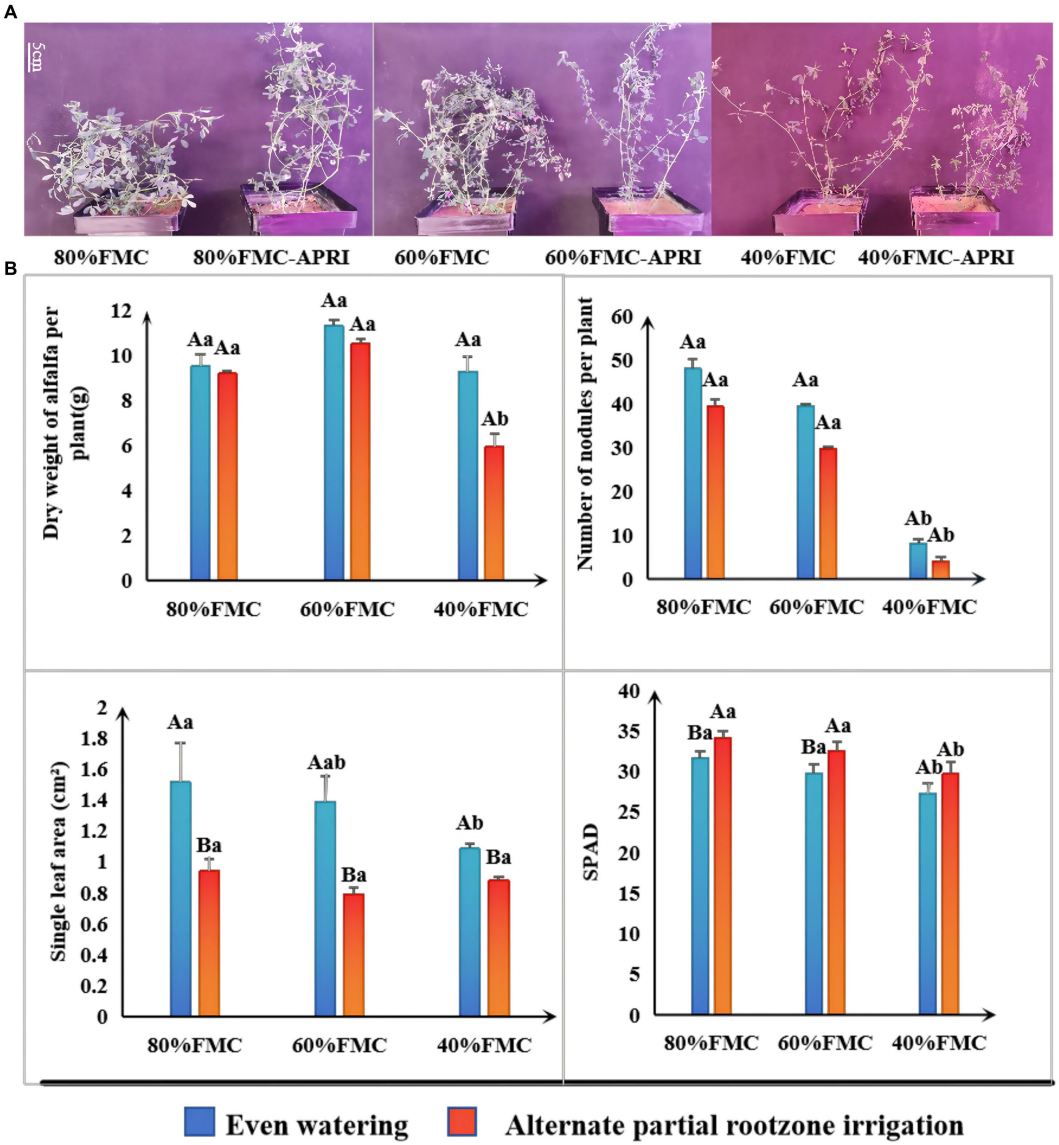
Growth response analysis of alfalfa plants treated with APRI (bars donate S.D.). Growth state of alfalfa at the initial flowering stage under each treatment **(A)**. Analysis of significant differences in growth indexes (dry weight, number of nodules, leaf area and SPAD) of alfalfa under different treatments **(B)**. The different capital letters indicate differences (*p* < 0.05) in each index of alfalfa treated with different watering methods under the same amount of watering. Different lowercase letters indicate differences (*p* < 0.05) in each index of alfalfa treated with the same watering method under different watering amounts.

The results of the significance analysis of the growth status and growth indices of single alfalfa plants under the different watering treatments are shown in Figure 2. With decreasing water supply, the number of nodules, chlorophyll content, and leaf area of the alfalfa plants under both watering methods exhibited decreasing trends. Under the different watering treatments, the dry weight and nodule number of alfalfa plants under even watering were greater than those under the APRI, but the difference was not significant. Similarly, the leaf area of alfalfa plants under even watering was significantly greater than that under APRI. Similarly, under the 80% FMC and 60% FMC watering treatments, the chlorophyll content of alfalfa plants under even watering was significantly lower than that under the APRI. Under even watering and alternate root zone watering, there was no significant difference in the various indices of alfalfa plants under the 80% FMC and 60% FMC watering treatments; however, under the 40% FMC watering treatment, the single-plant dry weight, chlorophyll content, and nodule number of alfalfa plants under the APRI, as well as the leaf area, chlorophyll content, and nodule number of alfalfa plants under even watering, were significantly lower than those under the other two watering treatments (Figure 2B).

### 3.2 The impact of the APRI on alfalfa rhizosphere microorganisms

#### 3.2.1 The impact of the APRI on rhizosphere MBC and MBN

**Figure 3:**
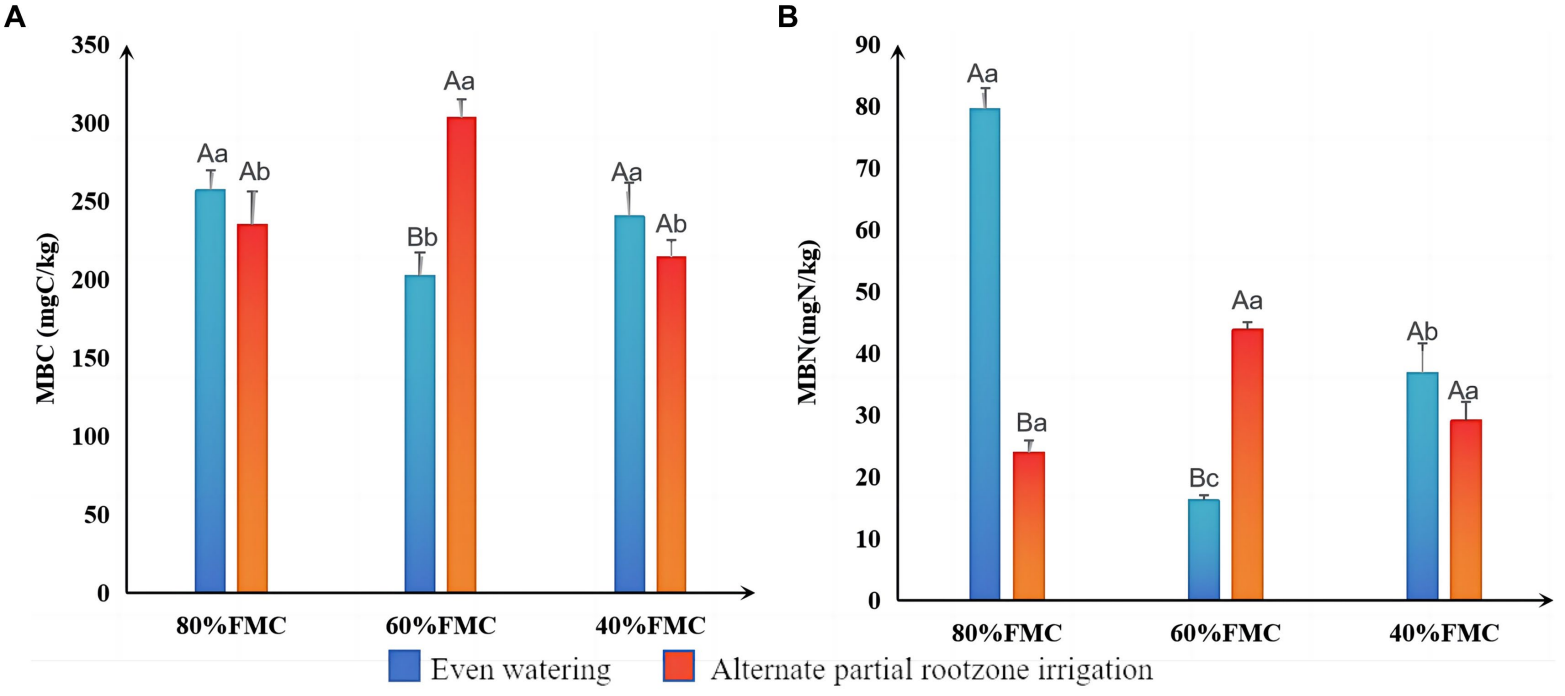
Significance analysis of MBC **(A)** and MBN **(B)** in the rhizosphere under different treatments (bars donate S.D.). The meanings of different capital letters and different lowercase letters are the same as that mentioned above.

The results of the differential analysis of MBC and MBN in alfalfa rhizosphere soil under different treatments are shown in Figure 3. The MBC and MBN under even watering at 80% FMC and 40% FMC were greater than those under the APRI treatment. However, at 60% FMC, the MBC and MBN in the even-watering treatment were significantly lower than those in the APRI treatment (*p* < 0.05).

#### 3.2.2 The impact of the APRI on the number of bacteria and fungi in the rhizosphere

**Figure 4:**
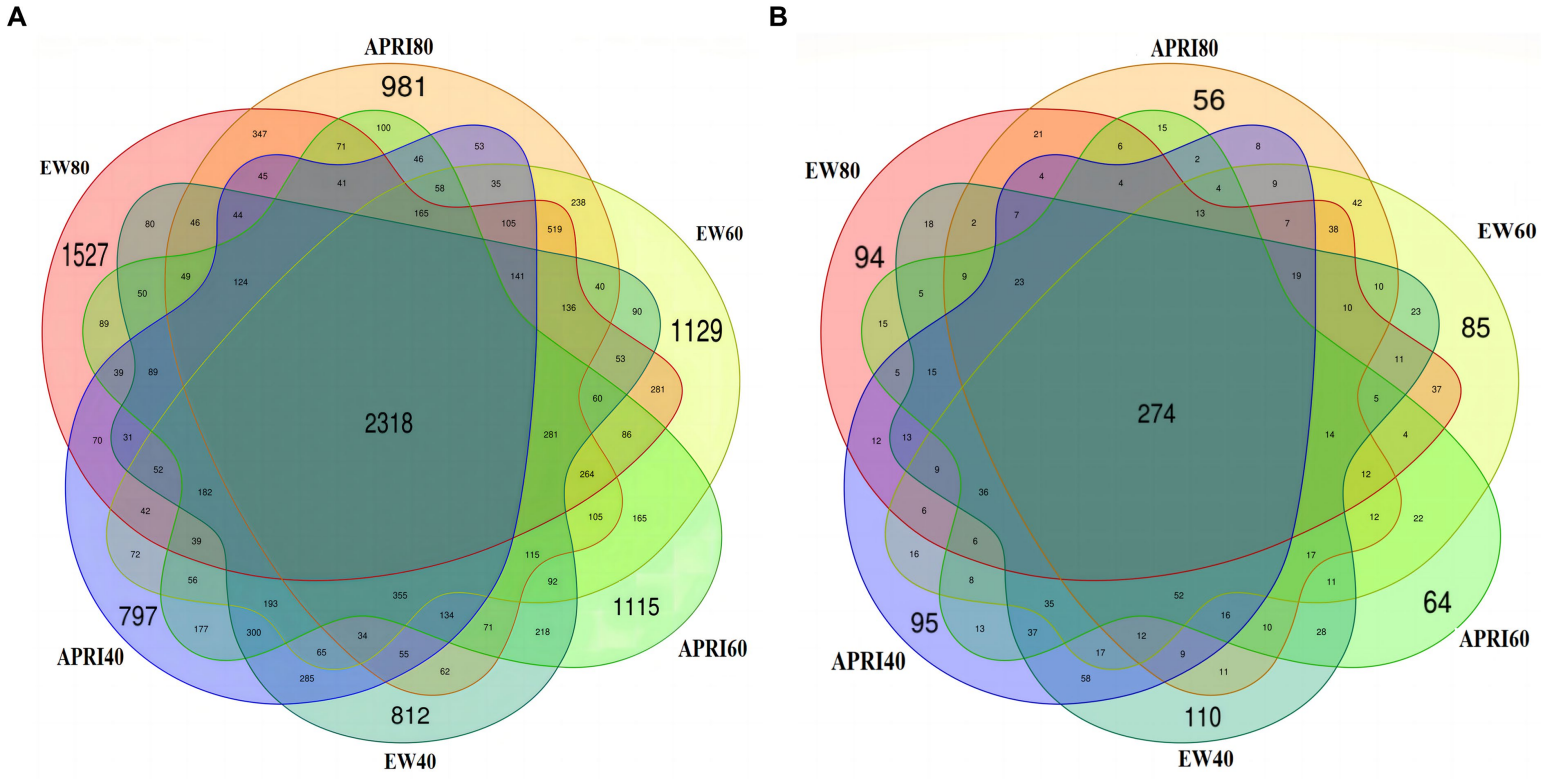
Venn map of rhizosphere soil bacteria **(A)** and fungi **(B)** under different treatments. EW80: The even watering amount is 80% FMC; APRI80: the watering amount of APRI is 80% FMC; EW60: the even watering amount is 60%; APRI60: the watering amount of APRI is 60% FMC; EW40: the even watering amount is 40% FMC; APRI40: the watering amount of APRI is 40% FMC.

The numbers of rhizosphere bacterial and fungal OTUs of alfalfa plants under the different treatments are shown in Figure 4. There were 2,318 total soil bacterial OTUs across all the treatments. The numbers of unique OTUs in the EW80, EW60, EW40, APRI80, APRI60, and APRI40 populations were 1,527, 1,129, 812, 981, 1,115, and 797, respectively (Figure 4A). The total number of soil fungal OTUs across all the treatments was 274. The numbers of unique OTUs in the EW80, EW60, EW40, APRI80, APRI60, and APRI40 populations were 94, 85, 110, 56, 64, and 95, respectively (Figure 4B). As the soil moisture decreased, the number of alfalfa root soil bacterial OTUs decreased under both irrigation treatments, while the number of fungal OTUs increased. Additionally, at the same watering level, the number of bacterial and fungal OTUs in the APRI treatment group was lower than that in the even-watering treatment group.

**Figure 5:**
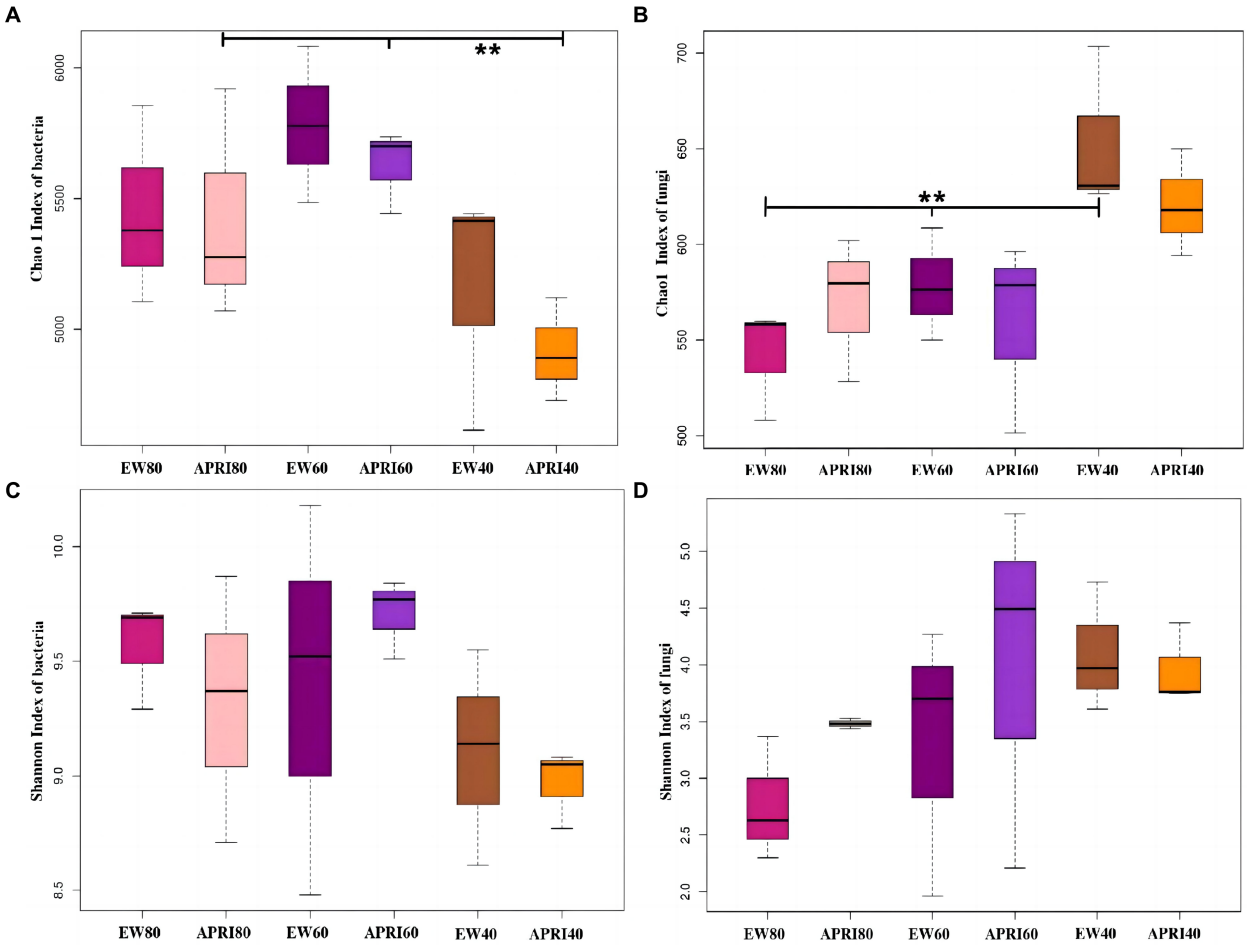
Alpha diversity index of rhizosphere soil bacteria and fungi under different treatments. Chao 1 indices of bacteria **(A)** and fungi **(B)** in the rhizosphere of the different treatment groups. And Shannon indices of bacteria **(C)** and fungi **(D)** in the rhizosphere of the different treatment groups. *p < 0.05, **p < 0.01. The meanings of APRI80-40 and EW80-40 are the same as that mentioned above.

The Chao1 index and Shannon index were used to analyze the alpha diversity of the rhizosphere bacterial community of alfalfa plants under the different treatments (Figure 5). In the APRI treatment, bacterial Chao1 index existed significant difference in the alfalfa rhizosphere under the different watering amounts, and the alfalfa rhizosphere bacterial Chao1 index under 60% FMC was significantly greater than that under 40% FMC (*p* < 0.01) (Figure 5A). In the even-watering treatment, the soil fungal Chao1 index varied significantly in the alfalfa rhizosphere and was significantly greater in alfalfa with 40% FMC than in alfalfa with a soil water content of 60% FMC or 80% FMC (*p* < 0.01) (Figure 5B). There was no significant difference in bacteria or fungi Shannon indices in the alfalfa rhizosphere soil between the different watering methods or different watering amounts (*p* > 0.05). In the even-watering treatment, as the water availability decreased, the Shannon index of bacteria in the rhizosphere of alfalfa significantly decreased (Figure 5C), while the Shannon index of the fungi gradually increased (Figure 5D). In the APRI treatment, with decreasing soil moisture content, the Shannon indices of both bacteria and fungi in the rhizosphere of alfalfa initially increased and then decreased. When the soil moisture content was 80% FMC or 40% FMC, the Shannon index of the bacterial community in the alfalfa rhizosphere under the APRI treatment was significantly lower than that under the even-watering treatment (Figure 5C). When the soil moisture content was 80% of the FMC or 60% of the FMC, the Shannon index of the soil fungi in the APRI treatment group was significantly greater than that in the even-watering treatment group (Figure 5D).

#### 3.2.3 The impact of APRI on the soil bacterial and fungal community structure in the rhizosphere

**Figure 6:**
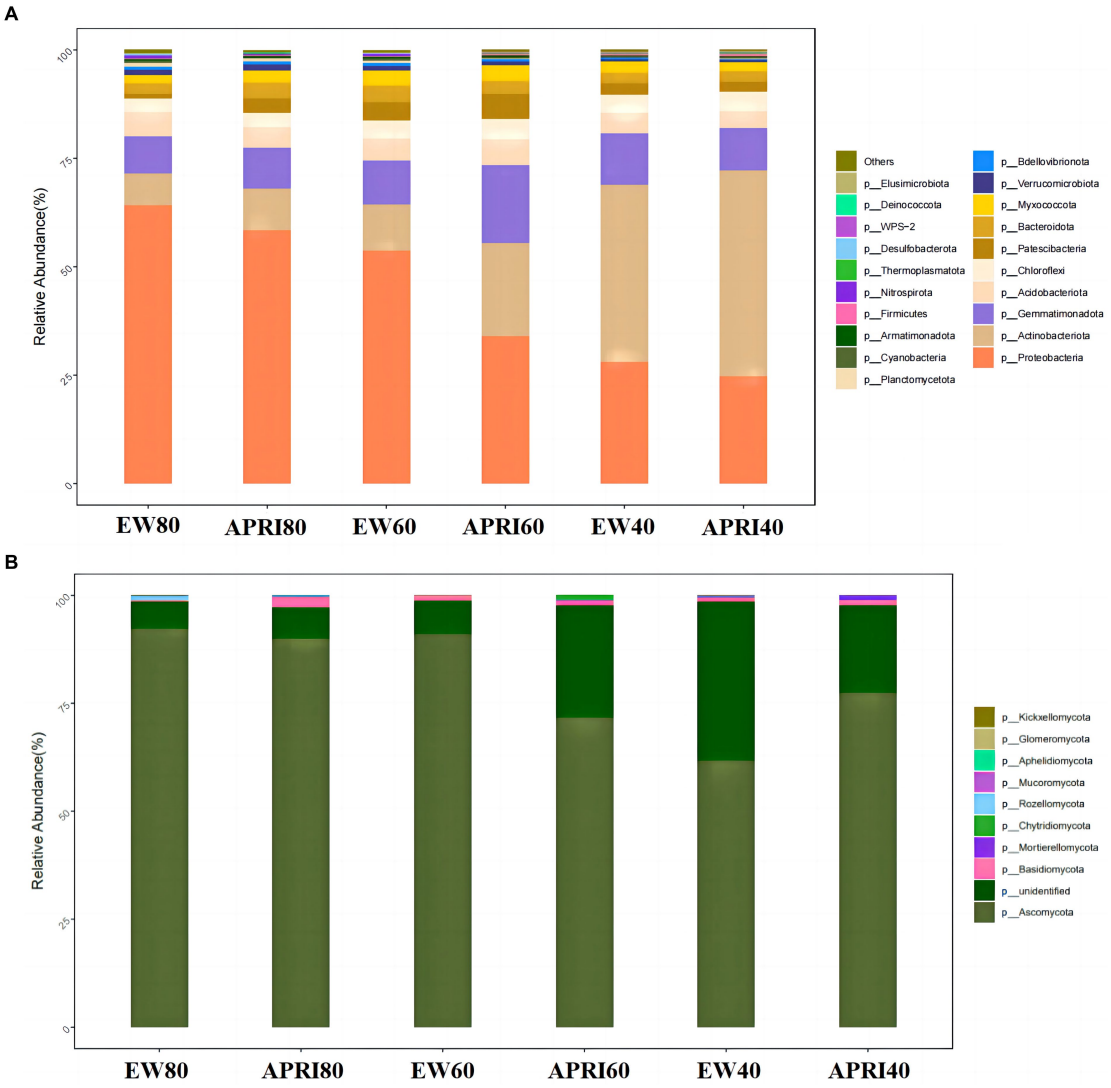
Relative abundances of bacteria **(A)** and fungi **(B)** in rhizosphere of different treatments at the phylum level. The meanings of APRI80-40 and EW80-40 are the same as that mentioned above.

The composition of bacterial and fungal taxa at the phylum level in the alfalfa rhizosphere under different treatments is shown in Figure 6. The phyla with relatively high abundances among the soil bacteria in each treatment included Proteobacteria, Actinobacteria, Gemmatimonadota, Acidobacteriota, Chloroflexi, Patescibacteria, Bacteroidota, and Myxococcota, which together accounted for 90% of the relative abundance of bacteria. Proteobacteria, Actinobacteria, Gemmatimonadota, and Acidobacteriota accounted for 80% of the total bacterial abundance, demonstrating their dominant status in the soil bacterial community (Figure 6A). The most abundant fungal phylum in the rhizosphere of each treatment was Ascomycota, which accounted for more than 60% of the relative fungal abundance and was the predominant fungal phylum in the soil. Additionally, the rhizosphere soil also contained fungi from the phyla Basidiomycota, Mortierellomycota, Mucoromycota, Aphelidiomycota, Glomeromycota, and Kickxellomycota (Figure 6B).

Under the same irrigation method, with decreasing watering, the relative abundance of Proteobacteria in the rhizosphere significantly decreased (*p* < 0.05), the relative abundance of Actinobacteria increased gradually, and the relative abundance of Basidiomycota fungi gradually decreased. Compared to that in the even-watering control group, the relative abundance of Proteobacteria in the APRI treatment group decreased, but the relative abundance of Actinobacteria increased. Of the three watering volume treatments, the APRI treatment affected the rhizosphere microorganisms the most at 60% FMC. At 60% FMC, the relative abundances of Ascomycota and Proteobacteria in the soil of the APRI treatment group were significantly lower than those in the even-watering treatment group (*p* < 0.05), and the relative abundances of Actinobacteria and unclassified K fungi in the alfalfa rhizosphere soil under the APRI treatment were significantly greater than those under the even-watering treatment (*p* < 0.05).

**Figure 7:**
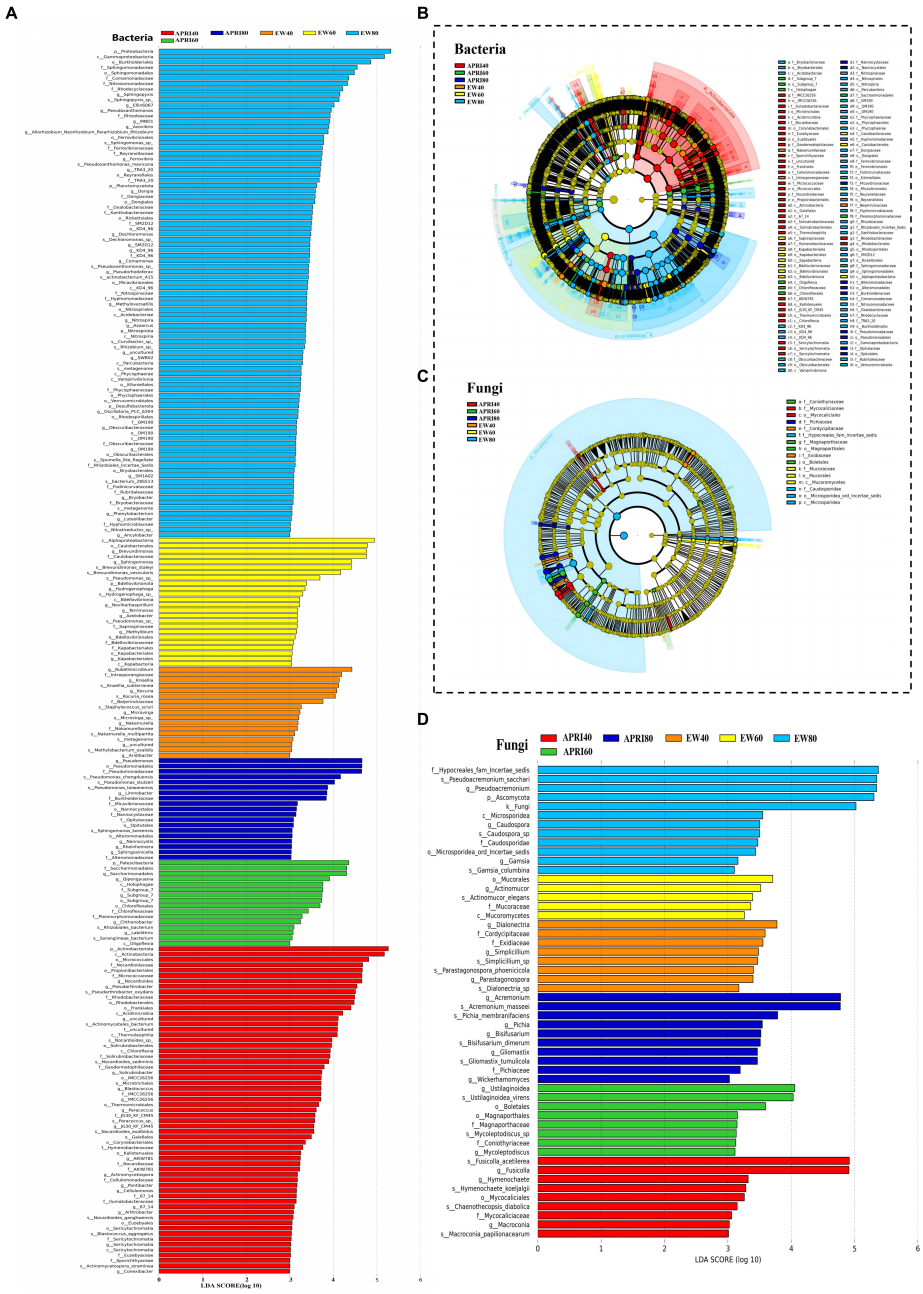
Results of LEfSe analysis of rhizosphere microorganisms of alfalfa under different treatments. Bar graph of bacteria **(A)** and fungi **(B)** LDA distribution from the LEfSe analysis based on taxonomic information. Show the significantly enriched microbial species under different treatments. The length of the histogram represents the enrichment degree of the enriched microorganisms. The longer the length, the greater the enrichment amount. Clade plots of bacteria **(C)** and fungi **(D)** evolution based on LEfSe analysis using taxonomic information. Circles radiating from inside to outside represent the taxonomic level from phylum to genus (or species), respectively. Each small circle at different taxonomic levels represents a classification at that level, and the small circle diameter size is directly proportional to the relative abundance size. Coloring principle: species with no significant differences are colored yellow, different species Biomarker follows the group, red nodes represent microbial taxa that play an important role in the red group, green nodes represent microbial taxa that play an important role in the green group, and other circles are similar. The species names indicated by the letters in English are shown in the legend to the right.

The LEfSe analysis of the alfalfa rhizosphere microorganisms under each treatment is shown in Figure 7. In the EW80, EW60, EW40, APRI80, APRI60, and APRI40 treatment groups, the alfalfa rhizosphere soil was significantly enriched in bacterial microorganisms, with 91, 24, 17, 19, 16, and 61 species, respectively (Figure 7A). There were 12, 5, 8, 10, 6, and 7 significantly enriched fungal species in the EW80, EW60, EW40, APRI80, APRI60, and APRI40 treatment groups, respectively (Figure 7B). Under even-watering treatment, the number of significantly enriched bacterial classes decreased with decreasing watering. Under the APRI treatment, the number of significantly enriched bacteria decreased, and the number of significantly enriched microorganisms in the alfalfa rhizosphere under 40% FMC was significantly greater than that in the alfalfa rhizosphere under 60% FMC and 80% FMC (Figure 7A). The number of fungi which was significantly enriched in alfalfa rhizosphere were significantly differed of different treatment groups (Figure 7B). There was no significant difference in the abundance of Alphaproteobacteria in the Proteobacteria in the rhizosphere soils of the different alfalfa treatment groups (*p* > 0.05) (Figure 7C). The enrichment of Mucorales in the alfalfa rhizosphere soil in the EW 60 treatment group was high and significantly differed from that in the other treatment groups (Figure 7D).

## 4 Discussion

This study revealed that the APRI treatment significantly increased the MBC and MBN compared to those under even-watering at 60% FMC. Moreover, under the APRI treatment, the rhizosphere MBC and MBN under the 60% FMC treatment were greater than those under the 40% FMC and 80% FMC treatments. MBC and MBN play important roles in the mineralization, humization and cyclic transformation of soil organic matter. A higher value indicates a greater number of microorganisms in the soil and a greater turnover capacity of nutrient elements (Liu H. et al., 2023). MBC and MBN are important parameters for evaluating soil quality changes (Pan et al., 2019). Therefore, the APRI +60% FMC treatment had the greatest amount of rhizosphere microorganisms and the greatest nutrient turnover ability. This may be because APRI causes the soil in the root area to undergo alternating drying and wetting, which not only provides the water needed for life activities but also results in good aeration conditions for the soil pores and provides beneficial living conditions for soil microorganisms (Wang et al., 2008). Soil rewetting may increase microbial abundance and activity, accelerating the mineralization rate of organic carbon (Denef et al., 2001). Dry–wet alternation may also cleave soil aggregates and increase the soil mineralization rate (Borken et al., 2003; Yv et al., 2008).

The Shannon index and Chao 1 index reflect the richness and evenness of species within the soil microbial communities. In this study, the bacterial Shannon index gradually decreased with increasing drought degree, which is consistent with previous findings that when soil is subjected to uniform drought stress, the abundance of soil microorganisms significantly decreases due to their relatively weak resistance to drought stress (Bastida et al., 2017; Upton et al., 2018; Vicente-Serrano et al., 2020; Fan et al., 2023). In the case of the APRI, the changes in the microbial diversity indices for both fungi and bacteria differed from those observed after even-watering, in which the Shannon index tended to increase initially and then decrease. However, the differences in the Shannon index between the treatments were not significant. The results of this study showed that under the APRI treatment, the largest rhizosphere bacterial Chao 1 index was observed for the 60% FMC treatment, and the Chao 1 index of the alfalfa rhizosphere with 60% FMC treatment was significantly greater than that of the alfalfa rhizosphere with 40% FMC treatment (*p* < 0.01). This may be because alternating dry and wet environments are beneficial for improving microbial diversity in the plant rhizosphere (Li et al., 2023). Some microorganisms die under drought conditions, after which the soil rehydrated, and the dead microorganisms are mineralized and release nutrients to support more microorganisms (Hamer et al., 2007; Xie et al., 2020). Rhizosphere microbial diversity is thus increased. Under 60% FMC, the APRI ensures both soil moisture and improved soil aeration (Schimel et al., 2007; Li et al., 2010), and this environment may be suitable for more microbial species. These findings also suggested that the APRI with 60% FMC is beneficial for enhancing the diversity of rhizosphere microbial communities.

Drought affects the soil environment, thereby influencing the diversity, composition, and structure of soil microorganisms (Wang et al., 2017). The rhizosphere bacteria are affected by drought and plant roots, and the changes in their composition and abundance are complex (Kichko et al., 2022). Previous research has shown that drought favors the enrichment of gram-positive bacteria, which replace gram-negative bacteria in the rhizosphere (Breitkreuz et al., 2021). The results of this study indicate that under reduced watering conditions, the relative abundance of Actinobacteria increased significantly in the alfalfa rhizosphere soil subjected to even watering and APRI, which is consistent with previous research findings. For example, drought induces the enrichment of actinomycetes in the microbial community of the sorghum (*Sorghum bicolor*) rhizosphere (Xu et al., 2021). This is because Actinomycetes have thicker peptidoglycan layers in their cell walls (Nocker et al., 2012) and can produce spores to resist unfavorable external factors (Boer et al., 2005), increasing their drought resistance. The relative abundance of actinomycetes in the rhizosphere soil in the APRI treatment was greater than that in the even-watering treatment under the same amount of water, possibly due to the increase in rhizosphere soil drought caused by the APRI treatment. Rhizobia addition may change the rhizosphere microbial population and community structure, but the results showed that rhizobia were not enriched in rhizosphere soil under the different treatments, and there was no significant difference in rhizobium abundance among the different treatments. This may be because the exogenous strains are less competitive in the rhizosphere, and drought stress has a significant negative effect on the survival of rhizobia (Sha et al., 2013). The growth rate of proteobacteria in rhizosphere soil is fast (Fierer et al., 2007), and they have thick cell walls to resist drought stress (Zheng et al., 2017). In a study on drought stress in sainfoin (*Onobrychis viciifolia*), drought stress significantly increased the relative abundance of Proteobacteria (Si et al., 2023). However, in the present study, the community relative abundance of Proteobacteria decreased, probably because APRI treatment increased the relative abundance of Actinomycetes, which increased the competitive advantage and subsequently inhibited the development and reproduction of Proteobacteria.

In the present study, the dominant fungus in the rhizosphere soil under each treatment was the phylum Ascomycota, which is consistent with the findings of previous studies on rhizosphere microbes in the Legaceae (Deng et al., 2022; Liu G. et al., 2023). Compared with bacteria, fungi have unique mycelial structures and more stable collinearity networks and are generally less affected by drought (Bapiri et al., 2010; de Vries et al., 2018). Under the 60% FMC treatment, the relative abundance of Ascomycota in the rhizosphere soil under APRI was significantly lower than that under even watering. However, the relative abundance of Ascomycota in the rhizosphere soil under the EW + 40%FMC treatment was not significantly different from that under the APRI+60%FMC treatment. These findings suggest that the APRI may exacerbate soil drought stress. However, the relative abundance of Ascomycota in the APRI+40%FMC treatment was greater than that in the EW + 40%FMC treatment. This may be because the soil of APRI +40% FMC was the most arid, leading to increased drought stress on alfalfa and increased root exudates (Walker et al., 2003; Jones et al., 2009), thereby increasing the abundance of Ascomycota in the rhizosphere soil (Zhang et al., 2018; Li et al., 2021). As drought severity increased, the relative abundance of unclassified K fungi in the alfalfa rhizosphere soil significantly increased, suggesting that these fungal taxa may be more drought-tolerant fungi hidden in the soil and may reorganize microbial structures in long-term arid soils.

## 5 Conclusion

At 80% FMC and 60% FMC, the APRI treatment had no significant impact on the growth or nodulation of alfalfa. However, at 40% FMC, the APRI significantly reduced the individual plant dry weight, chlorophyll content, and nodule number. The results suggest that an APRI with 60% FMC is a feasible water-saving alfalfa irrigation measure and provides a theoretical basis for water-saving alfalfa irrigation in arid and semiarid areas. But under 60% FMC + APRI treatment, the MBC and MBN of rhizosphere, relative abundance of Actinobacteria and unclassified K fungi and Chao 1 index of bacteria significantly increased, relative abundance of Ascomycetes and Proteobacteria in the alfalfa rhizosphere significantly reduced. 60%FMC + APRI treatment did significantly affect the groups, structure and diversity of the rhizosphere soil microbial communities. The microbial community structure is closely related to the soil ecosystem. The long-term impact of APRI treatment on alfalfa, a perennial grass, and the environment requires long-term monitoring tests.

## Data availability statement

The data presented in the study are deposited in the NCBI repository, accession number SRR29606239-SRR29606256 and SRR29600937-SRR29600954.

## Author contributions

JZ: Conceptualization, Data curation, Formal analysis, Investigation, Methodology, Visualization, Writing – original draft, Writing – review & editing. JX: Conceptualization, Data curation, Formal analysis, Investigation, Methodology, Writing – original draft. TW: Funding acquisition, Methodology, Project administration, Resources, Supervision, Validation, Writing – review & editing. QS: Methodology, Software, Writing – review & editing.
